# Treatment Choices in Women with Bipolar Disorder Seeking Pregnancy: A Clinical Case Illustration

**DOI:** 10.1155/2013/630732

**Published:** 2013-10-24

**Authors:** Corrado Barbui, Andrea Bertolazzi, Batul Hanife, Andrea Cipriani

**Affiliations:** WHO Collaborating Centre for Research and Training in Mental Health and Service Evaluation, Department of Public Health and Community Medicine, Section of Psychiatry, University of Verona, Policlinico G.B. Rossi, Piazzale Scuro 10, 37134 Verona, Italy

## Abstract

After ten years of successful maintenance treatment with lithium and olanzapine, a 40-year-old woman with bipolar disorder expressed concerns about continuing the use of medicines, as she was planning a pregnancy. In the past, she had suffered from five severe manic episodes with hospital admissions. After consultations with the treating psychiatrist, gynaecologist, and family doctor, olanzapine was stopped and lithium was gradually withdrawn. After few months, the patient, still in treatment with lithium 300 mg/die, experienced a new manic episode with hospital admission. Treatment with lithium and olanzapine was restored, and she progressively recovered. This case suggests that the risk of manic recurrence after ten years of maintenance treatment may be as high as the well-known risk of recurrence after few years of maintenance treatment, a consideration that doctors may find useful in the light of a complete absence of evidence on treatment choices after five years of successful maintenance treatment.

## 1. Introduction

This case highlights concerns around treatment choices in women with bipolar disorder seeking pregnancy [1]. While there is evidence that maintenance pharmacological treatment decreases the risk of recurrence, continuing treatment in women actively seeking a pregnancy bears a potential teratogenic risk for the foetus and is also associated with adverse effects. These concerns are particularly challenging in women who have been stable on maintenance treatment without manic or depressive episodes for several years, as after five years it becomes less clear if treatment continues to exert a protective effect [2]. Another challenge is that maintenance treatment with lithium may be particularly problematic to discontinue, with high rates of recurrence upon discontinuation [3].

## 2. Case Presentation

In March 2012, a 40-year-old woman with a long history of bipolar disorder discussed with the treating psychiatrist the possibility of planning a pregnancy. The patient had been free of manic episodes for ten years. She was on maintenance treatment with lithium 1,200 mg/die and olanzapine 2.5 mg/die and was worried about the potential teratogenic effects of lithium as well as the potential adverse effects of olanzapine during pregnancy. 

**Figure 1:**
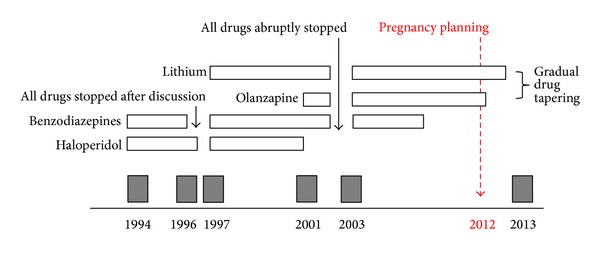
Psychiatric history of a 40-year-old woman with bipolar disorder. The gray box indicates hospital admission, and the white box indicates the length of pharmacological treatment.

In the past, she had suffered from five severe manic episodes that had always led to hospital admission (Figure 1). Each episode was characterised by persistent elevation of mood, increased energy and activity, psychomotor agitation, aggressiveness, a decreased need for sleep, marked distractibility, and flight of ideas. During all manic episodes, delusions were present, with marked loss of contact with reality. As frequently reported by the patient, manic episodes had always been associated with significant personal distress and social dysfunction, but, between episodes, a full recovery with normal functioning had always been achieved.

In terms of drug treatments, during the first two episodes, she was given haloperidol and benzodiazepines, and on discharge after the second admission, drug treatment was withdrawn as a result of a shared decision-making process (Figure 1). After a third manic relapse with hospital admission, lithium was added to haloperidol and benzodiazepines. This combination treatment was effective only in the short term, as in 2001 a new manic episode occurred during treatment (as documented by lithium blood concentration of 0.8 mEq/litre). Haloperidol was stopped and olanzapine started as an add-on treatment. In 2003, the patient abruptly stopped all drug treatments and subsequently suffered from a new episode with hospital admission. The same drug treatment was restored, and after recovery the patient agreed to see the treating psychiatrist on a weekly basis, in order to check lithium blood levels, treatment adherence, and mood. From 2003 onwards, the patient remained fully adherent to the therapeutic plan, lithium blood levels were stable at around 0.8-0.9 mEq/litre, mood remained stable, and a full and lasting recovery was achieved. Illness insight was high, and she was so frightened about the possibility of a new manic episode that the treating psychiatrist allowed her to call on his personal cell phone in case of emergency. In ten years, from 2003 to 2013, no calls had been received.

After initial talks with the treating psychiatrist about the possibility of actively seeking a pregnancy, during 2012, the patient had consultations with the treating gynaecologist and family doctor, who gave advice on pros and cons of stopping versus continuing drug treatment. Subsequent discussions involved the patient's partner, who had never seen the patient during a manic phase and was in favour of stopping all medicines. A decision was finally taken of stopping olanzapine and gradually withdrawing lithium, decreasing 150 mg every 15 days. Agreement was reached to keep weekly contacts with the treating psychiatrist.

The patient remained well until February 2013 when, still in treatment with lithium 300 mg/die, during a holiday in Rome, she started making phone calls to the treating psychiatrist, and the phone talks revealed that she was experiencing mood elevation, a progressive decreased need for sleep, marked distractibility, and increased energy and activity. Contact with reality did not appear to be lost. The patient's partner was advised to increase lithium and to take the patient back to Verona, where she was admitted to hospital, as it was impossible to manage this new manic episode in community. The patient spent more than two months in hospital and became psychotic, very aggressive, and agitated. Lithium was restored at 1,200 mg/die and olanzapine at 20 mg/die. At discharge, full recovery was achieved, although she felt sedated and mentally slowed down. Full-illness insight was rapidly restored.

## 3. Discussion

This case highlights the general clinical problem of the length of drug treatment in patients with bipolar disorder, who have been stable on maintenance therapy for several years, when valid reasons for discontinuation existed. The NICE guidelines recommend to continue treatment for up to five years if the person has risk factors for relapse, such as a history of frequent relapses or severe psychotic episodes [4, 5]. NICE recognises that the discontinuation of drugs might be necessary for a variety of reasons, including pregnancy, and in this case, gradual drug tapering is suggested. For lithium, NICE advises to gradually discontinue stepwise over at least four weeks and preferably over a longer period (up to 3 months). However, NICE has no guidance on therapeutic options after ten years of maintenance treatment, as no evidence is available after such a long period of time [2].

This case has implications for practicing doctors. First, we suggest that the risk of manic recurrence after ten years of maintenance treatment may be as high as the risk of recurrence after few years of maintenance treatment. Evidence exists suggesting that after an average length of 30 months of lithium maintenance treatment, the risk of manic episode following discontinuation translates into a 28-fold difference for patients on and just off lithium [3]. Here, we suggest that such a staggering increased risk may be maintained in the very long term. Second, patients on maintenance treatment with lithium may be particularly vulnerable to recurrence, as compared with patients on maintenance treatment with other mood stabilisers, as lithium itself may induce a sensitivity state so that discontinuation may be particularly problematic [3]. Here, a manic episode was observed so close with lithium decrease, and before discontinuation, that one may hypothesise that it was not a spontaneous recurrence as in untreated bipolar illness but rather an induced recurrence in a bipolar illness sensitised to more than ten years of lithium exposure. Third, the common belief that lithium withdrawal in stable patients may carry the additional risk of subsequent lithium refractoriness may not be true when lithium is combined with other therapies, as in this case, lithium and olanzapine were reintroduced with clinically appreciable beneficial effects. 

In terms of research priorities, we argue that studies should gently switch from current strong focus on short-term outcomes in acutely ill patients to the more challenging issue of treatment choices in individuals exposed to psychotropic drugs in the long term.
